# Workplace ostracism influences hotel employees pro-environmental behaviour through green work engagement

**DOI:** 10.1038/s41598-026-38569-6

**Published:** 2026-02-09

**Authors:** Huang Haijiang, Muhammad Rafiq

**Affiliations:** UCSI Graduate Business School, UCSI University, Kuala Lumpur, Malaysia

**Keywords:** Workplace ostracism, Employees’ pro-environmental behaviour, Green management initiatives, Environmental passion, Hotel industry, China, Environmental social sciences, Psychology, Psychology

## Abstract

This study examines how workplace ostracism influences hotel employees’ pro-environmental behavior, highlighting the psychological and organizational conditions that shape sustainable actions in the hospitality sector. Guided by social cognitive theory, the model tests green work engagement as a mediating mechanism and green management initiatives and environmental passion as boundary conditions. Data were collected through a structured questionnaire administered to 528 employees from four- and five-star hotels in major Chinese cities. Using Hayes’ PROCESS macro, the study assessed both mediation and moderated effects. Results show that workplace ostracism significantly reduces employees’ pro-environmental behavior, and this relationship is partially mediated by green work engagement. Moreover, green management initiatives strengthen the negative association between workplace ostracism and green work engagement, while environmental passion strengthens the effect of green work engagement on pro-environmental behavior. These findings contribute to sustainability and hospitality research by demonstrating how interpersonal exclusion undermines environmentally responsible behavior and by identifying organizational practices and individual motivations that can intensify or enhance these effects.

## Introduction

Pro-environmental behavior has become a strategic priority for organizations seeking to improve environmental sustainability while maintaining operational excellence^1^. This issue is particularly salient in the hospitality industry, where the delivery of services is resource intensive and employees’ day-to-day behaviors directly influence energy consumption, waste reduction, and environmental performance^2^. As a result, the ability of hotel employees to voluntarily engage in environmentally friendly actions greatly contributes to organizational sustainability outcomes^3^. Prior research has identified several positive antecedents including recognition, rewards, leadership support, and intrinsic motivation that encourage employees to adopt green behaviors^4–6^. However, the workplace is also shaped by negative social experiences, and much less is known about how these adverse conditions discourage employees from participating in sustainability-related activities. This overlooked dimension is crucial, because sustainable practices often require voluntary effort beyond formal job duties.

Workplace ostracism, defined as the experience of being ignored or excluded by coworkers or supervisors, represents one such negative social condition^7^. It has been consistently linked to reduced psychological well-being and diminished discretionary efforts across different organizational settings^8^. Although research has examined its influence on employees’ sense of belonging and self-esteem^9^, very few studies have explored whether ostracism undermines pro-environmental behavior a form of discretionary, prosocial behavior that benefits the organization and its stakeholders^10^. This omission is particularly problematic in hospitality contexts, where frequent interpersonal interaction and intense teamwork increase employees’ vulnerability to social exclusion. Prior evidence indicates that ostracized employees tend to withdraw from extra-role actions, display emotional detachment, or reduce cooperation^11,12^. These reactions strongly suggest that pro-environmental behavior may also decline when employees experience social exclusion. Yet, despite the theoretical relevance, the relationship between workplace ostracism and employees’ environmentally responsible actions remains empirically underexplored.

Understanding this relationship requires unpacking both the internal psychological mechanisms and the contextual factors that shape employees’ responses to social exclusion. Green work engagement a state in which employees feel energetic, dedicated, and absorbed in sustainability-related tasks may serve as a key psychological pathway through which ostracism reduces pro-environmental behavior^13^. When employees feel excluded, their motivation to contribute voluntarily to sustainability initiatives may weaken, resulting in lower green engagement. At the same time, organizational and personal contexts can buffer or intensify these effects. Green management initiatives, which represent structured environmental practices and policies signaling that sustainability is a core organizational value^14^, may help maintain employee engagement even when social interactions are strained. Likewise, environmental passion employees’ emotional attachment to environmental protection^15^ may motivate individuals to continue acting sustainably regardless of interpersonal challenges. However, empirical evidence on how these mechanisms and boundary conditions jointly shape pro-environmental behavior under workplace ostracism remains limited.

To address these gaps, this study investigates the direct effect of workplace ostracism on hotel employees’ pro-environmental behavior, the mediating role of green work engagement, and the moderating influences of green management initiatives and environmental passion. By examining a large sample of employees working in four- and five-star hotels across major Chinese cities, this study provides context-specific insights into how social exclusion interacts with both organizational practices and personal motivations to influence sustainability-related behavior. This integrated approach enables a more comprehensive understanding of when and why employees continue or fail to engage in environmentally responsible actions in socially challenging work environments.

This research makes several important contributions. *First*, it extends pro-environmental behavior literature by examining workplace ostracism as a negative antecedent, thereby shifting attention beyond the predominantly positive predictors commonly emphasized in sustainability research. *Second*, it contributes to psychological scholarship by identifying green work engagement as a key mechanism linking social exclusion to employees’ sustainable behavior, highlighting how diminished engagement can translate into reduced environmental actions. *Third*, drawing on the principles of social cognitive theory, the study demonstrates how organizational cues (green management initiatives) and personal motivations (environmental passion) jointly shape employees’ behavioral responses to ostracism. This theoretical positioning offers a multi-level explanation that integrates individual, interpersonal, and contextual drivers. This study advances both sustainability and hospitality management literature by clarifying how adverse workplace experiences influence environmentally beneficial behavior.

## Theoretical underpinning and hypothesis development

### Social cognitive theory

Social cognitive theory (SCT) provides a rigorous framework for understanding how individuals interpret workplace experiences and regulate their behaviors through the interaction of personal, behavioral, and environmental determinants^16,17^. SCT proposes a “triadic reciprocal causation” system, wherein personal cognitions, environmental cues, and enacted behaviors continuously influence one another^16,17^. Within this framework, workplace ostracism represents a powerful negative environmental cue that can undermine core personal factors such as psychological safety, perceived social value, and self-efficacy. Self-efficacy the belief in one’s capability to perform and influence outcomes is central to SCT and is highly sensitive to social feedback^18^. When employees perceive exclusion or interpersonal neglect, these experiences diminish perceived competence and weaken motivation to undertake behaviors requiring voluntary effort. Because pro-environmental behavior in organizations is discretionary and often exceeds formal job expectations, a decline in self-efficacy and social reinforcement may substantially reduce employees’ willingness to engage in sustainability-related actions. Thus, SCT provides a strong theoretical grounding for anticipating that workplace ostracism will negatively influence pro-environmental behavior.

SCT also emphasizes the importance of internal self-regulatory processes such as motivation, engagement, and goal-directed cognition in determining behavior. These processes explain how green work engagement can operate as a mediating mechanism between workplace ostracism and pro-environmental behavior. Engagement reflects an individual’s internalized sense of purpose, focus, and energetic involvement in tasks, which aligns closely with SCT emphasis on intrinsic regulation and personal agency^19,20^. When employees experience ostracism, their diminished self-efficacy^21^ and impaired social belonging may erode their engagement in sustainability-related activities. Conversely, employees who remain psychologically engaged in green tasks are more likely to initiate and sustain pro-environmental behavior because they perceive personal meaning and competence in such actions. In SCT terms, green work engagement represents the personal cognitive and motivational processes that translate environmental cues (ostracism) into behavioral outcomes (green actions). Thus, the mediating role of green work engagement directly reflects SCT view that individuals’ internal psychological states govern how they respond to social experiences.

Furthermore, SCT underscores that environmental conditions and personal dispositions shape how cognitive processes translate into behavior. Green management initiatives exemplify the environmental determinants highlighted in SCT specifically modeling, reinforcement, and normative cues^22^. When organizations provide visible and consistent green policies, they create strong situational signals that pro-environmental behavior is valued, thereby strengthening employees’ motivation even when interpersonal experiences (such as ostracism) are negative. These cues can buffer the damaging effects of ostracism by maintaining clarity around organizational expectations and enhancing perceived environmental efficacy. On the personal side, environmental passion reflects SCT “personal determinant” dimension, which includes internalized motivation, value alignment, and emotional investment^23^. Employees with high environmental passion are more likely to sustain pro-environmental behavior because intrinsic commitment increases persistence despite adverse social conditions. Consequently, green management initiatives and environmental passion function as environmental and personal moderators precisely the type of boundary conditions emphasized in SCT reciprocal causation model. By integrating these moderators, the study applies SCT at multiple levels, explaining the complex interplay between social experiences, internal motivation, and sustainability behavior.

### Relationship between workplace ostracism and pro-environmental behaviour

Workplace ostracism refers to situations where employees feel ignored, excluded, or socially disregarded by coworkers or supervisors^24^. These experiences carry significant emotional and cognitive weight, often signaling to employees that they are not valued members of the work environment. Pro-environmental behaviour, on the other hand, consists of voluntary, discretionary actions aimed at reducing environmental harm and supporting organizational sustainability efforts, such as recycling, conserving energy, and responsibly using resources^25^. Although substantial research has examined the negative consequences of ostracism such as reduced well-being, performance, and cooperation^26^ little empirical research has explored whether social exclusion also undermines employees’ willingness to engage in environmentally responsible behaviour. Considering that pro-environmental behaviour is a form of prosocial, extra-role contribution, it is plausible that negative interpersonal experiences weaken employees’ motivation to participate in such voluntary practices.

From a SCT perspective, workplace ostracism can disrupt core personal cognitions that regulate behaviour. A distinctive SCT mechanism relevant here is cognitive appraisal of social cues, which refers to employees’ interpretation of how their environment evaluates them^16^. When employees perceive neglect or exclusion, they may interpret these cues as indicators that their contributions formal or voluntary are undervalued^27^. This negative appraisal can trigger emotional withdrawal^27^, reduce perceived agency, and weaken moral responsibility toward collective goals. Importantly, this SCT lens differs from traditional self-efficacy explanations: the emphasis here is not on capability beliefs, but on how employees evaluate the worthiness of contributing within an environment that appears socially rejecting. If employees feel they do not belong or that their efforts will go unnoticed, they are less likely to persist in discretionary environmental actions that require personal initiative. Thus, the appraisal of social rejection forms a pathway through which workplace ostracism can diminish pro-environmental behaviour.

#### H1

Workplace ostracism is negatively related to pro-environmental behaviour.

### Relationship between workplace ostracism and green work engagement

Workplace ostracism disrupts employees’ connection to their work environment by signaling interpersonal rejection and reduced social value^28^. Existing research shows that such exclusion erodes general work engagement, as employees begin to feel psychologically detached and less motivated to invest in their roles^29^. Extending this understanding to sustainability initiatives, green work engagement employees’ energetic, dedicated, and absorbed involvement in environmental tasks^13^ is similarly vulnerable to the emotional and motivational strain triggered by social exclusion. Employees who feel marginalized may experience a breakdown in their sense of belonging and significance within the organization^30^, making it less likely for them to allocate the additional cognitive, emotional, and physical effort required to engage in voluntary sustainability practices. Because green work engagement typically exceeds formal job requirements, the motivational cost of taking initiative becomes particularly high for individuals already coping with social rejection.

A distinct mechanism within SCT helps clarify this relationship: self-regulatory resource depletion. SCT posits that sustaining purposeful action requires continuous self-regulation, including monitoring, goal prioritization, and emotional management^19^. When employees face ostracism, they expend substantial self-regulatory resources managing negative emotions^31^, making sense of ambiguous social cues, and navigating strained interpersonal relationships. This drain on personal regulatory capacity limits the resources available for initiating and sustaining focused engagement in environmental tasks. Unlike the self-efficacy-focused explanation in other contexts, the emphasis here is on how social exclusion depletes the internal regulatory systems that energize and maintain engagement. Moreover, ostracism weakens access to social reinforcement and constructive feedback critical environmental supports in SCT further reducing employees’ ability to stay motivated and committed to sustainability initiatives. Thus, ostracism undermines both the personal and environmental components necessary for green work engagement.

#### H2

Workplace ostracism is negatively related to green work engagement.

### Relationship between green work engagement and pro-environmental behaviour

Growing evidence suggests that employees who are energetically involved in sustainability-related tasks are more likely to take initiative in environmentally responsible behaviours^32^. Karatepe, et al. ^13^, for example, found that green work engagement is positively associated with pro-environmental behaviour among hospitality employees, highlighting the motivational role of engagement in driving voluntary green actions. Yet, despite these insights, theoretical explanations for this link remain relatively narrow, leaving opportunities to deepen understanding of the mechanisms that connect employees’ psychological involvement in sustainability tasks to their actual environmental behaviours at work.

A distinct lens from SCT helps illuminate this relationship, observational learning combined with goal alignment. SCT posits that employees actively learn and internalize patterns of behaviour by observing environmental cues, role models, and organizational signals^16^. When employees are highly engaged in green tasks, they pay closer attention to sustainability norms, observe colleagues’ environmental efforts more closely, and internalize these behaviors as part of their own goal system. Engagement heightens awareness of environmental standards and enhances the salience of sustainability-related goals^33^. This mechanism is different from cognitive appraisal and self-regulatory depletion. So, the emphasis is on how engaged employees actively model, adopt, and replicate environmentally responsible practices in response to organizational cues and peer behaviors. As employees become more absorbed in green activities, they cognitively align themselves with environmental values, increasing the likelihood of translating engagement into concrete pro-environmental actions.

Furthermore, SCT highlights that engaged employees are more likely to see a clear connection between their behavior and desirable environmental outcomes a process known as outcome expectancy^34^. This expectancy strengthens the motivational force that turns engagement into action. In the hospitality context, where sustainability efforts are increasingly visible and integrated into daily operations, such alignment between personal engagement and environmental goals becomes especially powerful. Through this combination of observational learning, goal alignment, and positive outcome expectancy, green work engagement fosters a stronger inclination toward pro-environmental behaviour.

#### H3

Green work engagement is positively related to pro-environmental behaviour.

### Mediating role of green work engagement on the relationship between workplace ostracism and pro-environmental behaviour

Workplace ostracism disrupts employees’ social connection with colleagues and supervisors, often leading to emotional strain, role disengagement, and reduced willingness to participate in activities beyond their formal responsibilities^29^. Pro-environmental behaviour, which relies heavily on voluntary extra-role efforts, is especially sensitive to such disruptions^35^. At the same time, green work engagement employees’ energetic and dedicated involvement in sustainability-related tasks has been found to strongly predict pro-environmental actions in hospitality settings^13^. Taken together, these patterns suggest that workplace ostracism may indirectly diminish pro-environmental behaviour by undermining the psychological engagement necessary for employees to participate actively in organizational sustainability initiatives.

A distinctive mechanism within SCT helps clarify this mediating effect: the disruption of the self-regulation cycle and motivational displacement. SCT asserts that personal behaviour is guided through an iterative self-regulation process in which individuals set goals, monitor progress, and adjust their efforts accordingly^16,19^. When employees experience workplace ostracism, this self-regulatory cycle becomes disrupted. The emotional and cognitive demands of coping with social exclusion displace attention away from sustainability goals, weaken internal motivation, and reduce the personal agency required to sustain engagement in green tasks. As a result, employees allocate fewer psychological resources to environmental responsibilities, leading to diminished green work engagement. Reduced engagement then lowers the likelihood of engaging in pro-environmental behaviours because employees no longer perceive themselves as active contributors to sustainability efforts. Through this lens, green work engagement becomes the crucial conduit through which the negative environmental cue of ostracism translates into weakened pro-environmental behaviour.

#### H4

Green work engagement mediates the relationship between workplace ostracism and pro-environmental behaviour.

### Moderating role of green management initiatives

Employees who experience workplace ostracism often report diminished motivation and weakened engagement because exclusion signals that their contributions are unrecognized or undervalued^36^. In sustainability contexts, these feelings can particularly undermine green work engagement, which requires initiative, discretionary effort, and psychological commitment^37^. However, organizations vary substantially in the clarity and strength of their environmental policies^38^. Green management initiatives which encompass structured policies, resource allocation, leadership support, and visible sustainability practices may offer a compensatory mechanism that encourages engagement even among employees who feel socially marginalized^14^. When such initiatives are strong, sustainability goals are clearly communicated, resourced, and reinforced; this may help employees remain engaged with green tasks even when interpersonal conditions are unfavourable.

SCT provides a distinct mechanism for explaining this moderating effect, compensatory environmental structuring. SCT argues that when one part of the triadic system (personal, behavioral, or environmental factors) becomes impaired, other components can compensate to maintain functioning^16^. Workplace ostracism disrupts the personal system by damaging belongingness, emotional stability, and self-regulatory capacity^39^. However, strong green management initiatives can provide counterbalancing environmental cues clear norms, structured expectations, visible modelling from leaders, and task guidance that restore behavioural motivation despite weakened personal conditions^14^. This logic differs from observational learning and self-regulation arguments used in earlier hypotheses: here, the emphasis is on how a structured pro-environmental context substitutes for missing social support, offering employees predictable signals and reinforcement that sustain green work engagement. Thus, when green management initiatives are strong, the negative effect of ostracism on engagement should be weaker because employees rely on organizational structures rather than interpersonal acceptance to guide their behaviour.

#### H5

Green management initiatives moderate the relationship between workplace ostracism and green work engagement.

### Moderating role of environmental passion environmental passion

Green work engagement reflects employees’ psychological investment in sustainability-related tasks and has been shown to encourage pro-environmental behaviour in service settings^13^. However, employees vary greatly in how strongly they identify with environmental values, meaning that engagement does not always translate into behaviour with equal intensity. Environmental passion defined as intense, positive feelings and personal enthusiasm toward environmental protection^15^ represents a particularly influential personal disposition that can strengthen the behavioural consequences of engagement. Employees with high environmental passion derive meaning, purpose, and emotional satisfaction from sustainability activities^15^; therefore, when they are engaged in green work tasks, they are more likely to extend their involvement beyond formal job requirements and display consistent, voluntary pro-environmental behaviours^40^. Conversely, employees with low environmental passion may engage in green tasks out of compliance rather than conviction, resulting in weaker translation of engagement into actual behaviour.

SCT provides a clear mechanism for why environmental passion amplifies the impact of engagement on pro-environmental behaviour. SCT emphasizes that behaviour is guided not only by environmental cues but also by self-regulatory processes built around personal values and intrinsic motivations^16^. For highly passionate employees, environmental protection forms part of their internal standards and self-identity. When these employees engage in sustainability-related work, they experience intrinsic rewards such as pride, moral fulfilment, and alignment with personal ideals^41^. These internal reinforcements strengthen self-efficacy and behavioural consistency, making it more likely that engagement results in observable pro-environmental action. In contrast to earlier hypotheses where SCT mechanisms centred on environmental cues or social feedback this moderator operates through deeply internalized values that energize and sustain behavioural follow-through. Thus, environmental passion enhances the personal determinant component within SCT triadic reciprocal causation, increasing the likelihood that green work engagement is converted into sustained pro-environmental behaviour.

#### H6

Environmental passion moderates the relationship between green work engagement and pro-environmental behaviour.

## Methods

### Sample and procedures

Data for this study were collected from employees working in four- and five-star hotels located in major Chinese cities, including Beijing, Shanghai, and Hangzhou. These cities were selected due to their concentration of upscale hotels and well-established sustainability practices, providing a suitable context for examining workplace ostracism and pro-environmental behaviour. Data collection was carried out over a four-month period, from November 2024 to February 2025, ensuring adequate coverage of operational cycles within the hospitality sector. A structured self-administered questionnaire was distributed using the online survey platform Sojump (www.sojump.com), which is widely used in China and enables efficient data management and rapid distribution. Following the recommendation of Cavana, et al.^42^ to target respondents who are best positioned to provide relevant information, the study employed a purposive sampling strategy, focusing exclusively on hotel employees in the intended industry segment.

To ensure that only eligible participants proceeded with the survey, several screening questions were placed at the beginning of the questionnaire. These items verified that respondents were currently employed in four- or five-star hotels and held positions relevant to daily operational activities. The survey link was disseminated through hospitality-related professional networks, industry forums, and WeChat groups commonly used by hotel employees. This dissemination strategy enhanced the likelihood that respondents were genuinely affiliated with the target sector, thereby improving sample relevance and representativeness. Participation was voluntary, and respondents were informed that their responses would remain confidential.

To accommodate respondents’ language preferences and reduce comprehension bias, the questionnaire was offered in both English and Chinese. A rigorous back-translation procedure was used to ensure linguistic accuracy and cultural appropriateness^43^. First, a bilingual translator translated the English questionnaire into Chinese. A second independent translator then translated this version back into English. Any discrepancies identified were reviewed and resolved by an expert panel fluent in both languages. This approach ensured that the final Chinese version accurately reflected the original meaning and intent of the items.

An introductory cover letter accompanied the questionnaire, outlining the purpose of the study, ensuring anonymity, and clarifying that the data would be used strictly for academic research. A total of 553 responses were initially collected. A thorough data-cleaning process was subsequently conducted to remove incomplete or invalid submissions. Following Hair Jr, et al.^44^, responses with extensive missing data were eliminated to reduce potential threats to validity; as a result, 25 questionnaires were excluded. The final dataset comprised 528 valid responses.

The demographic profile of the respondents reflects diversity across key characteristics. Educational levels ranged from High School (16.9%) and undergraduate degrees (34.8%) to Master’s degrees (39.8%), M.Phil (7.8%), and PhD qualification (0.8%). Regarding marital status, 28.8% of respondents were single and 71.2% were married. Age distribution showed that 16.9% were aged 25 or below, 42.2% were between 26 and 35, 23.1% were 36–45, 11.7% were 46–55, and 6.1% were 56 or older. Gender representation was balanced, with 46% female and 54% male respondents. This demographic distribution provides a robust and heterogeneous sample for examining the proposed relationships.

### Measures

The study employed well-established measurement scales drawn from prior empirical research to ensure content validity and conceptual clarity. Pro-environmental behaviour was assessed using the scale developed by Bissing-Olson, et al.^45^, which captures discretionary, sustainability-oriented actions undertaken by employees in workplace settings. Green management initiatives were measured using items adapted from Luu^46^, reflecting the extent to which organizations implement formal environmental practices, policies, and support systems. Green work engagement was assessed using the scale by Aboramadan^47^, which evaluates employees’ energy, dedication, and absorption in sustainability-related tasks. Workplace ostracism was measured using the widely validated instrument developed by Ferris, et al.^24^, which captures employees’ perceptions of being ignored or excluded by colleagues or supervisors. Finally, environmental passion was measured using items adopted from Robertson and Barling^48^ used in their study.

All items were rated using a five-point Likert scale ranging from 1 (Strongly Disagree) to 5 (Strongly Agree). This response format is commonly used in organizational behaviour research and supports consistent interpretation across respondents. To enhance transparency and replicability, the complete set of measurement items for each construct is presented in Appendix A of the revised manuscript.

### Data analysis

Data analysis was conducted using SPSS 26 and AMOS 24 following a structured, multi-stage approach. First, descriptive statistics were computed to profile the sample and examine the distributional characteristics of the data. Internal consistency of the constructs was assessed through Cronbach’s alpha, ensuring that all scales demonstrated acceptable reliability levels. Subsequently, confirmatory factor analysis (CFA) was performed in AMOS to validate the measurement model. CFA was chosen because it provides a rigorous assessment of construct validity, including factor loadings, model fit indices, convergent validity, and discriminant validity. This step ensured that the latent constructs were empirically distinct and aligned with theoretical expectations.

Following validation of the measurement model, hypothesis testing was conducted using Hayes’ PROCESS macro for SPSS, a widely used regression-based tool for mediation, moderation, and conditional process analysis^49,50^. PROCESS was selected because it offers a robust regression-based framework for testing mediation, moderation, and conditional indirect effects without requiring manual computation of interaction terms or bias-corrected confidence intervals. Specifically, Model 4 was used to examine the mediating effect of green work engagement on the relationship between workplace ostracism and pro-environmental behaviour, while Model 21 was applied to test the dual moderating effects of green management initiatives and environmental passion. These models were complemented by 5,000 bootstrap samples, allowing for precise estimation of indirect and interaction effects, as recommended by contemporary mediation–moderation literature.

### Confirmatory factor analysis

CFA was conducted using AMOS 24 following the two-step approach recommended by Anderson and Gerbing^51^, which involves validating the measurement model prior to testing the structural relationships. The proposed five-factor model comprising workplace ostracism, green management initiatives, green work engagement, environmental passion, and pro-environmental behaviour demonstrated a satisfactory fit to the data. The model yielded the following fit statistics: χ^2^ = 1961.899, df = 918, CFI = 0.93, TLI = 0.93, RMSEA = 0.04, all of which fall within widely accepted thresholds for good model fit. The SRMR value was 0.05, further supporting the adequacy of the measurement model.

To establish discriminant validity, the hypothesized five-factor model was compared against a series of alternative models in which conceptually distinct constructs were successively combined. As shown in Table 1, the proposed model outperformed all alternative specifications, including the four-factor, three-factor, two-factor, and one-factor models. Chi-square difference tests confirmed that the five-factor model provided significantly better fit relative to more constrained models, thereby demonstrating that each construct captured unique variance and should be treated as empirically distinct. These results support the validity of the measurement structure and indicate that the observed indicators load appropriately on their respective latent variables.

**Table 1:** Confirmatory factor analysis.

Models	χ^2^	df	CFI	TLI	RMSEA
Five-factor model (WOS, GMI, ENP, GWE, PEB)	1961.899	918	0.93	0.93	0.04
Four-factor model (WOS, GMI, ENP, GWE + PEB)	2422.314	922	0.90	0.90	0.05
Three-factor model (WOS, GMI, ENP + GWE+PEB)	4797.306	925	0.74	0.74	0.08
Two-factor model (WOS, GMI + ENP+GWE + PEB)	6558.176	927	0.64	0.62	0.10
One-factor model (WOS + GMI+ENP + GWE+PEB)	7042.756	930	0.61	0.58	0.11

Note: *n* = 528; workplace ostracism = WOS; green management initiatives = GMI; green work engagement = GWE; environmental passion = ENP; pro-environmental behaviour = PEB.

Source(s): Authors’ own creation.

### Validity test

Convergent and discriminant validity were assessed using established criteria recommended by Black, et al.^52^. Convergent validity was evaluated through factor loadings, average variance extracted (AVE), and composite reliability (CR). As shown in Table 2, all standardized factor loadings exceeded the recommended threshold of 0.70, and all CR values ranged from 0.80 to 0.95, demonstrating strong internal consistency. The AVE for each construct was above 0.50, indicating that the items captured a substantial proportion of variance in their respective latent variables. These results collectively confirm adequate convergent validity for all five constructs.

Discriminant validity was assessed using the Fornell–Larcker criterion, which requires that the AVE for each construct exceed both the maximum shared variance (MSV) and the average shared variance (ASV). As presented in Table 2, all AVE values were greater than corresponding MSV and ASV values, supporting discriminant validity across all constructs. In addition, a supplementary HTMT assessment was conducted, and all HTMT ratios were below the conservative threshold of 0.85, further confirming that each construct is empirically distinct.

To examine potential multicollinearity, the study also assessed tolerance and variance inflation factor (VIF) values. Tolerance values ranged from 0.66 to 0.73 (above the 0.10 cutoff), and VIF values ranged from 1.36 to 1.50 (well below the threshold of 10). These results indicate the absence of multicollinearity concerns and further support the robustness of the measurement model. Together, these validity assessments confirm that the study’s measures demonstrate strong convergent and discriminant validity and that multicollinearity was not an issue in the dataset.

**Table 2:** Convergent and discriminant validity.

Scale	Items	Factor Loading	α	CR	AVE	MSV	ASV
Workplace ostracism	WOS-1	0.75	0.92	0.92	0.55	0.22	0.16
	WOS-2	0.76					
	WOS-3	0.73					
	WOS-4	0.70					
	WOS-5	0.80					
	WOS-6	0.74					
	WOS-7	0.71					
	WOS-8	0.75					
	WOS-9	0.73					
	WOS-10	0.72					
GMI	GMI-1	0.83	0.95	0.95	0.61	0.31	0.20
	GMI-2	0.80					
	GMI-3	0.85					
	GMI-4	0.71					
	GMI-5	0.76					
	GMI-6	0.74					
	GMI-7	0.80					
	GMI-8	0.77					
	GMI-9	0.76					
	GMI-10	0.75					
	GMI-11	0.84					
	GMI-12	0.81					
	GMI-13	0.70					
GWE	GWE-1	0.86	0.93	0.93	0.70	0.30	0.18
	GWE-2	0.84					
	GWE-3	0.87					
	GWE-4	0.78					
	GWE-5	0.82					
	GWE-6	0.84					
Environmental passion	ENP-1	0.72	0.93	0.93	0.58	0.19	0.13
	ENP-2	0.76					
	ENP-3	0.79					
	ENP-4	0.78					
	ENP-5	0.74					
	ENP-6	0.76					
	ENP-7	0.73					
	ENP-8	0.71					
	ENP-9	0.79					
	ENP-10	0.79					
Pro- environmental behaviour	PEB-1	0.70	0.80	0.80	0.57	0.22	0.15
	PEB-2	0.79					
	PEB-3	0.76					

Note: *n* = 528; Workplace ostracism = WOS; green management initiatives = GMI; green work engagement = GWE; environmental passion = ENP; pro-environmental behaviour = PEB; AVE = Average variance extracted; CR = Composite reliability; ASV = Average shared variance; MSV = Maximum shared variance.

Source(s): Authors’ own creation.

### Common method bias

Given that the data for this study were collected through self-reported questionnaires, several procedural and statistical techniques were employed to minimize and assess the potential impact of common method bias (CMB). Following the recommendations of^53^, multiple ex-ante procedural remedies were incorporated. Participation was voluntary, and respondents were assured of anonymity and confidentiality through the introductory cover letter, which reduces evaluation apprehension and socially desirable responding. The items were drawn from well-established and validated scales, and the questionnaire layout randomized item order to minimize consistency motifs and reduce response patterning.

In addition to these procedural safeguards, several ex-post statistical techniques were applied. First, Harman’s single-factor test was conducted using an unrotated principal components analysis. The results showed that no single factor accounted for the majority of the variance, indicating that CMB was unlikely to be driven by a dominant common factor. Second, a common latent factor (CLF) approach was evaluated within the CFA framework. The inclusion of a latent method factor did not significantly improve model fit, and the differences between standardized loadings with and without the CLF were below the recommended 0.20 threshold, suggesting that method effects were minimal.

To complement these tests, full collinearity VIFs were computed in line with Kock^54^. All latent variables exhibited VIF values below threshold value, providing further evidence that common method variance does not pose a substantial threat to the results. These techniques provide a more rigorous assessment than reliance on mediation or moderation tests, as recommended in recent methodological literature.

## Results

### Descriptive analysis

Table 3 shows descriptive and correlation data. The data showed a significant negative association between workplace ostracism and several variables: pro-environmental behaviour (*r* = -.35, *p* < .01), green work engagement (*r* = -.45, *p* < .01), green management initiatives (*r* = -.47, *p* < .01), and environmental passion (*r* = -.32, *p* < .01), aligning with anticipated trends. Furthermore, Table 3 also reveals a significant positive relationship between green work engagement and pro-environmental behaviour, with a correlation coefficient of (*r* = .24, *p* < .01).

**Table 3:** Correlation analysis.

Variables	1	2	3	4	5
1. Workplace ostracism	-				
2. Pro environmental behaviour	− 0.351^**^	-			
3. Green work engagement	− 0.450^**^	0.248^**^	-		
4. Green management initiatives	− 0.471^**^	0.385^**^	0.358^**^	-	
5. Environmental passion	− 0.326^**^	0.346^**^	0.478^**^	0.344^**^	–

Note: *n* = 528; p** < 0 0.01; p* < 0 0.05.

Source(s): Authors’ own creation.

### Direct effects and mediation analysis (PROCESS model 4)

The direct and indirect effects in the proposed mediation model were examined using Hayes’ PROCESS Macro Model 4 with 5,000 bootstrap samples (*n* = 528). Table 4 presents the coefficients for the direct paths, the indirect effect, and the total effect. The analysis first examined the total effect of workplace ostracism on pro-environmental behaviour (i.e., without the mediator). The results showed a significant negative total effect (B = − 0.87, SE = 0.10, 95% CI [–1.0660, − 0.6740]), supporting H1, indicating that higher workplace ostracism is associated with lower pro-environmental behaviour.

Next, workplace ostracism significantly and negatively predicted green work engagement (B = − 0.86, SE = 0.07, 95% CI [–0.9972, − 0.7228]), supporting H2. Green work engagement also positively predicted pro-environmental behaviour (B = 0.14, SE = 0.06, 95% CI [0.0224, 0.2576]), supporting H3. The indirect effect of workplace ostracism on pro-environmental behaviour through green work engagement was significant (B = − 0.12, SE = 0.07; Boot 95% CI [–0.2761, − 0.0010]). Because the bootstrapped confidence interval does not include zero, green work engagement mediates the relationship between workplace ostracism and pro-environmental behaviour. After including the mediator, the direct effect of workplace ostracism on pro-environmental behaviour remained significant (B = − 0.75, SE = 0.11, 95% CI [–0.9656, − 0.5344]), indicating partial mediation and supporting H4.

**Table 4:** Results for mediation analysis.

Effect	B	SE	95% CI
Workplace ostracism → Green work engagement	-0.86	0.07	-0.9972, -0.7228
Workplace ostracism → Pro environmental behavior (direct path)	-0.75	0.11	-0.9656, -0.5344
Green work engagement → Pro environmental behavior	0.14	0.06	0.0224, 0.2576
Indirect effect (WO → GWE → PEB)	-0.12	0.07	-0.2761, -0.0010
Direct effect (WO → PEB controlling GWE)	-0.75	0.11	-0.9656, -0.5344
Total effect (WO → PEB without GWE)	-0.87	0.1	-1.0660, -0.6740

Note: *n* = 528; SE = Standard error; CI = Confidence interval; Workplace ostracism = WOS; green work engagement = GWE; pro-environmental behaviour = PEB.

Source(s): Authors’ own creation.

### Moderation analysis (PROCESS model 21)

The moderating effects of green management initiatives and environmental passion were tested using PROCESS Model 21, which allows simultaneous estimation of moderation at both stages of the mediation model. The results are summarized in Table 5.

**Table 5:** Results for moderation analysis.

Variables	Beta values	SE	CI (95%)
Green work engagement			
Workplace ostracism	-0.68	0.08	− 0.8501, − 0.5201
Green management initiatives	0.25	0.04	0.1635, 0.3412
Workplace ostracism × Green management initiatives	-0.26	0.07	− 0.3985, − 0.1179
Pro environmental behavior			
Green work engagement	0.12	0.07	0.0689, 0.1335
Environmental passion	0.31	0.05	0.2204, 0.4192
Green work engagement × Environmental passion	0.13	0.04	0.0514, 0.2091

Note: *n* = 528; SE = Standard error; CI = Confidence interval.

Source(s): Authors’ own creation.

**Fig. 1:**
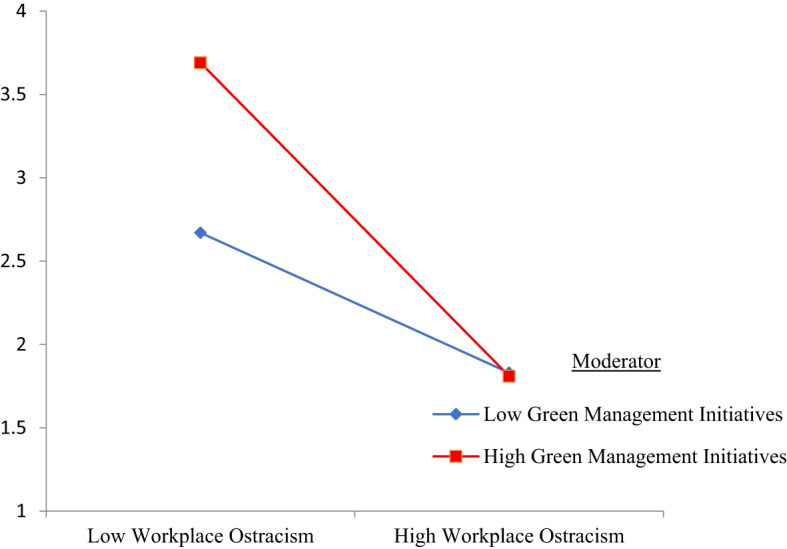
Moderating role green management initiatives. Source(s): Authors’ own creation.

The interaction between workplace ostracism and green management initiatives was significant and negative (B = − 0.26, SE = 0.07, 95% CI [–0.3985, − 0.1179]), indicating that the negative effect of workplace ostracism on green work engagement becomes stronger when green management initiatives are higher. Specifically, the negative slope becomes less severe when green management initiatives are strong, meaning that supportive organizational environmental practices help buffer the detrimental effects of ostracism on employees’ engagement with green tasks. This provides empirical support for H5. The interaction pattern is illustrated in Fig. 1.

**Fig. 2:**
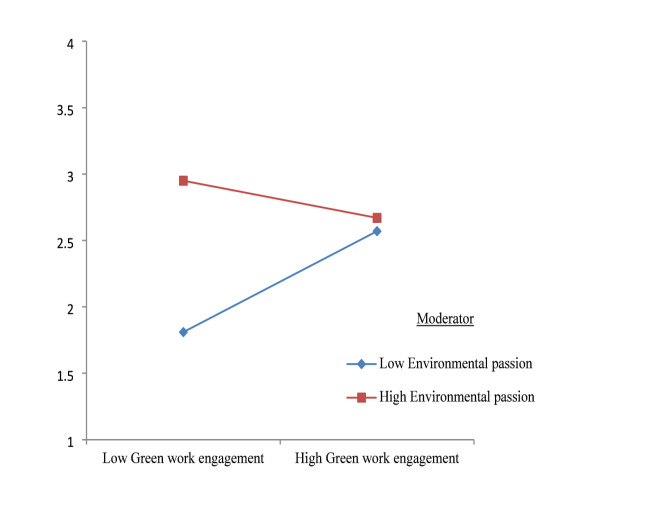
Moderating role of environmental passion. Source(s): Authors’ own creation.

Similarly, the interaction between green work engagement and environmental passion was significant (β = 0.13, SE = 0.04, *p* < .05; 95% CI = 0.0514, 0.2091). This finding shows that environmental passion strengthens the positive association between green work engagement and pro-environmental behaviour, supporting H6. Employees with higher environmental passion translate their engagement into actual pro-environmental actions more effectively than those with lower passion. This interaction is depicted in Fig. 2.

## Discussion

The present study examined how workplace social dynamics shape pro-environmental behaviour among hotel employees in China. Grounded in SCT^16^, the findings highlight how workplace ostracism, employees’ psychological engagement, and both organizational and personal factors jointly influence sustainable actions in hospitality settings. In a context where pro-environmental behaviour is increasingly central to hotels’ sustainability and competitive positioning^1,3^, the results provide nuanced evidence on how social exclusion and motivational processes affect employees’ willingness to contribute to green initiatives.

Consistent with H1, workplace ostracism showed a significant negative association with pro-environmental behaviour. This aligns with prior evidence that ostracism undermines employees’ psychological well-being, belongingness, and discretionary work efforts^9^. In hospitality settings where employees play a direct role in implementing energy-saving practices, waste reduction, and other environmental routines^2^, feeling ignored or excluded by colleagues or supervisors can reduce employees’ motivation to invest in extra-role green actions. The present study extends earlier work that mainly linked ostracism to reduced cooperation and performance^11^ by showing that its detrimental effects also encompass environmentally responsible behaviour—an outcome that is critical for hotels striving to improve sustainability performance.

Support for H2 further demonstrates that workplace ostracism significantly reduces green work engagement. This finding is consistent with the view that exclusion signals a lack of social value and weakens employees’ psychological connection to their work roles^28^. From an SCT perspective, employees rely on social cues, feedback, and supportive interactions to sustain motivation and confidence^16^. When employees feel ignored or marginalized, they lose access to the reinforcement and modelling needed to remain energized and absorbed in sustainability-related tasks. The results therefore position green work engagement as a socially embedded construct shaped not only by individual pro-environmental orientations but also by the quality of everyday workplace relationships and the presence or absence of inclusive social dynamics.

Hypothesis 3 was also supported, showing that employees with higher green work engagement are more likely to display pro-environmental behaviour. This is consistent with hospitality research demonstrating that engaged employees are more inclined to perform discretionary, sustainability-oriented behaviours^32^. SCT suggests that engagement strengthens outcome expectations and self-regulatory processes, increasing the likelihood that employees enact behaviours aligned with organizational environmental goals^19^. In hotel operations—where sustainable practices depend on consistent frontline actions rather than one-off initiatives—the positive link between engagement and pro-environmental behaviour underscores the strategic value of strengthening green work engagement as a psychological resource.

The mediation findings support H4 by showing that green work engagement partially explains how workplace ostracism influences pro-environmental behaviour. Specifically, ostracism reduces employees’ engagement with sustainability tasks, which in turn lowers their likelihood of engaging in pro-environmental actions. This clarifies the psychological process through which social exclusion undermines sustainable behaviour, extending prior work that documented ostracism’s negative outcomes without fully unpacking the underlying mechanisms^35^. Interpreted through SCT, ostracism disrupts self-regulatory cycles and shifts attention away from green goals^19^, leading to reduced green work engagement and, consequently, weaker environmental contributions. The mediation pathway therefore highlights engagement as a key conduit linking social barriers to environmental outcomes in hospitality workplaces.

Hypothesis 5 was supported, showing that green management initiatives moderate the relationship between workplace ostracism and green work engagement. The interaction term was negative, indicating that the adverse effect of ostracism on engagement is stronger when green management initiatives are higher. In hotels where environmental expectations and sustainability practices are highly visible and strongly emphasized^38^, ostracized employees may perceive a sharper mismatch between organizational demands and their reduced social standing. Rather than experiencing green systems as supportive, they may experience heightened pressure and intensified exclusion, which accelerates disengagement. Consistent with SCT, when social reinforcement is absent, stronger performance and value demands can heighten strain and perceived inadequacy^39^, magnifying the impact of ostracism on engagement. This suggests that strong green systems cannot substitute for healthy interpersonal climates and should be implemented alongside inclusion-oriented practices.

Finally, H6 was supported, showing that environmental passion moderates the relationship between green work engagement and pro-environmental behaviour. The relationship was stronger for employees with higher environmental passion. Passionate employees possess stronger intrinsic motivation, internalized environmental values, and self-regulatory capacity—mechanisms SCT identifies as central to personal agency and value-based self-regulation^19^. For these employees, sustainability is closely linked to self-identity, meaning that engagement in green tasks more readily translates into consistent and meaningful pro-environmental actions^40^. In contrast, employees with lower environmental passion may engage in green activities more instrumentally, resulting in a weaker conversion of engagement into behaviour. This finding highlights the value of cultivating environmental passion to amplify the behavioural pay-off of green work engagement and strengthen employee-driven sustainability efforts in hotels.

The findings underline that sustainability behaviour in hotels is not merely a function of environmental policies or technical systems but is deeply rooted in employees’ social experiences, motivational states, and internal values. Workplace ostracism poses a significant barrier to sustainable action, and the evidence suggests that highly emphasized green management initiatives may, under conditions of exclusion, intensify disengagement rather than offset it. At the same time, environmental passion strengthens the translation of green work engagement into pro-environmental behaviour. By connecting ostracism, engagement, green management initiatives, and environmental passion within an SCT framework, the study deepens understanding of how social and psychological mechanisms jointly shape pro-environmental behaviour in hospitality contexts.

### Theoretical contributions

The findings of this research make several noteworthy contributions to theory on workplace dynamics, employee engagement, and pro-environmental behaviour in organizational settings. First, the study extends the application of SCT by demonstrating that negative workplace experiences specifically workplace ostracism can impede employees’ engagement in sustainability-related behaviour. SCT has often been used to explain how positive environmental cues, learning opportunities, and supportive feedback foster desirable behaviour^16,17,22^. By positioning workplace ostracism as a detrimental environmental cue that signals rejection and low social value^9^, this study shows that the “environmental” component in SCT also includes adverse social signals that disrupt employees’ willingness to enact discretionary green behaviours. This moves beyond prior work that mainly highlights enabling social conditions and demonstrates that SCT can equally account for how hostile or exclusionary climates suppress sustainability-related action.

Second, the study identifies green work engagement as a key psychological mechanism through which workplace ostracism influences pro-environmental behaviour, thereby enriching both engagement and sustainability literatures. Prior research has shown that engagement energizes employees to contribute extra-role and climate-friendly behaviours in hospitality settings^13^. Our findings add a critical nuance: green work engagement is itself vulnerable to social exclusion and functions as the conduit through which ostracism undermines pro-environmental behaviour. Interpreted through SCT, this mediation illustrates how disrupted self-regulation and diminished motivational focus^19^ translate negative social cues into weaker environmental behaviours. Conceptually, this positions green work engagement not only as a positive driver of sustainability outcomes but also as a fragile resource that can be eroded by unfavourable workplace dynamics.

Third, the study advances theory by showing that both organizational-level and individual-level moderators shape the process through which workplace ostracism translates into sustainability behaviour. The moderating roles of green management initiatives and environmental passion concretely illustrate SCT principle of triadic reciprocal determinism, whereby personal factors, environmental structures, and behaviour continually co-influence one another^19^. Green management initiatives represent formal environmental cues policies, practices, and leadership signals that structure expectations around sustainability, while environmental passion reflects deeply internalized, value-based motivation for environmental protection. By demonstrating that these two types of moderators alter the strength of the ostracism–engagement and engagement–behaviour links, the study emphasizes that pro-environmental behaviour emerges from multilevel interactions rather than from isolated antecedents.

Finally, by integrating workplace ostracism, green work engagement, green management initiatives, and environmental passion within a unified SCT framework, the study offers a more holistic theoretical account of how employees construct environmentally responsible behaviour in real organizational contexts. Rather than treating pro-environmental behaviour as the outcome of either structural policies^2^ or individual dispositions alone, the findings support a dynamic view in which social context, internal motivational states, and organizational sustainability systems reciprocally shape behaviour. This multi-layered perspective enriches theoretical conversations in sustainability and hospitality management by framing employee pro-environmental behaviour as the product of ongoing interactions between workplace social experiences, psychological engagement, and both organizational and personal pro-environmental orientations.

#### Practical implications

The findings of this research offer several important practical implications for managers and policymakers in the hospitality sector, where employee-driven sustainability practices are essential for reducing resource consumption, strengthening environmental performance, and enhancing service quality^3^. First, the results clearly show that workplace ostracism significantly undermines employees’ pro-environmental behaviour. Given that hotel employees frequently collaborate and interact with guests, even subtle exclusion can diminish motivation, belongingness, and willingness to engage in discretionary green actions^8^. Hospitality organizations should therefore prioritize building inclusive, socially supportive workplaces by establishing clear anti-ostracism policies, confidential reporting systems, and regular communication or teamwork workshops to prevent interpersonal exclusion and strengthen social cohesion.

Second, the mediating role of green work engagement emphasizes the need for hotels to actively cultivate psychological engagement in sustainability-related tasks. Engaged employees are more likely to contribute voluntary pro-environmental actions, especially in service-intensive contexts. Managers can enhance engagement by involving employees in designing or implementing green initiatives such as recycling systems, waste-sorting procedures, energy-saving campaigns, or guest-facing eco-programmes. Providing autonomy and recognizing employee contributions can further reinforce their sense of purpose and meaning in sustainability work.

Third, the moderating role of green management initiatives demonstrates that organizational structures and environmental cues strongly influence how employees respond to interpersonal dynamics. Hotels should therefore institutionalize sustainability through formal policies, clear environmental standards, leadership modelling, and visible green practices. Strong organizational signals for example, green certifications, eco-labels, environmentally responsible purchasing, or resource-efficient technologies reinforce that sustainability is a core value of the organization. Importantly, the findings suggest that structured sustainability systems must be accompanied by supportive social environments, as strong green expectations may intensify the negative psychological effects of ostracism when social support is lacking.

Finally, the moderating effect of environmental passion reveals that employees with strong intrinsic environmental motivation are more likely to translate engagement into consistent pro-environmental behaviour^40^. Hospitality organizations can cultivate environmental passion by offering sustainability awareness workshops, environmental education, employee volunteering programmes, or partnerships with local environmental organizations. These activities help employees internalize environmental values, deepen emotional connection with ecological issues, and foster stronger personal motivation to act sustainably at work. These implications highlight the need for a dual focus on social inclusion and environmental commitment. Reducing workplace ostracism, cultivating green engagement, institutionalizing sustainability practices, and nurturing environmental passion can create a workplace ecosystem where pro-environmental behaviour becomes a natural, consistent part of employees’ daily activities. Such strategies will not only enhance environmental performance but also contribute to an inclusive, motivated, and resilient workforce within the hospitality industry.

### Limitations and future directions

Despite the meaningful insights generated by this study, several limitations warrant consideration. First, the data were collected exclusively from employees in four- and five-star hotels in China, which may restrict the generalizability of the findings to other regions with different cultural norms, organizational structures, and sustainability expectations. Workplace ostracism is known to manifest differently across cultural contexts particularly when comparing collectivist Asian societies with more individualistic Western regions such as Europe or the United States potentially altering its impact on employee behaviour. Similarly, pro-environmental behaviour is shaped by national environmental policies and cultural attitudes toward sustainability, meaning that results from the Chinese hospitality sector may not fully translate to other global settings. Additionally, the study relied on self-reported measures, which may introduce response biases, despite the use of procedural and statistical remedies. Future research should incorporate multi-source data, behavioural observations, or objective performance records to overcome these limitations.

Future studies may also broaden the conceptual model by incorporating additional factors that influence the relationship between workplace ostracism and pro-environmental behaviour. Variables such as leadership style, organizational culture, environmental climate, and employee psychological well-being may offer deeper insights into the mechanisms that drive sustainable behaviour at work. Longitudinal research designs would be valuable for examining how the effects of ostracism evolve over time, as cross-sectional data limit causal interpretation. Moreover, cross-cultural comparative studies for example, comparing hospitality employees across Europe, North America, and East/Southeast Asia would help determine whether the strength and direction of these relationships vary across different socio-cultural environments. Experimental or intervention-based research could also test strategies aimed at reducing the negative effects of ostracism while strengthening pro-environmental engagement. Together, such extensions would enhance theoretical understanding and provide more globally relevant guidance for fostering sustainable practices in diverse organizational settings.

## Conclusion

This study examined how workplace ostracism influences pro-environmental behaviour among hotel employees in China, highlighting the mediating role of green work engagement and the moderating effects of green management initiatives and environmental passion. The findings show that social exclusion undermines employees’ engagement in sustainability initiatives, and this detrimental effect becomes stronger when green management initiatives are higher. In contrast, environmental passion strengthens the positive effect of green work engagement on pro-environmental behaviour, indicating that employees with stronger environmental passion translate engagement into sustainable actions more effectively. By integrating social cognitive theory, the study offers a multi-level perspective on how individual, social, and organizational factors interact to shape sustainable behaviour at work. These insights contribute to hospitality and sustainability research and provide actionable implications for managers seeking to strengthen employee engagement in environmental practices. This study underscores the importance of fostering inclusive workplaces and implementing green initiatives in ways that do not inadvertently intensify pressure or disengagement among ostracized employees, while also nurturing employees’ environmental passion to support long-term environmental responsibility.

## Data Availability

The datasets generated and analyzed during the current study are available from the corresponding author upon reasonable request.
